# Supplementary material for: Apathy-Related Symptoms Appear Early in Parkinson’s Disease
Source: Healthcare (Basel). 2022 Jan 4;10(1):91. doi: 10.3390/healthcare10010091 (PMC8775593; doi:10.3390/healthcare10010091)
Supplement: Supplementary file 1 [file healthcare-10-00091-s001.zip › healthcare-1420526-supplementary.pdf]

Table S1. Comparison of Outcome variables between the Mild group and Moderate group.

	Apathy (MDS-UPDRS 1.5)	Fatigue (MDS-UPDRS 1.13)	Loss of Pleasure (BDI-II.4)	Loss of Interest in People or Activities (BDI-II.12)	Loss of Energy (BDI-II.15)	Loss of Interest in Sex (BDI-II.21)
Whole Sample	0.546 ± 0.946	1.59 ± 0.951	0.808 ± 0.737	0.575 ± 0.694	1.13 ± 0.484	0.840 ± 0.930
Mild (1-2)	0.408 ± 0.888	1.43 ± 0.861	0.634 ± 0.681	0.479 ± 0.652	1.04 ± 0.429	0.629 ± 0.765
Moderate (2.5=4)	0.75 ± 1.0	1.848 ± 1.03	1.06 ± 0.747	0.714 ± 0.736	1.27 ± 0.531	1.14 ± 1.06

Means and standard deviations reported for the apathy construct and apathy-related construct items presented in table 3.
